# Importance of Nutrient Availability and Metabolism for Skeletal Muscle Regeneration

**DOI:** 10.3389/fphys.2021.696018

**Published:** 2021-07-16

**Authors:** Jamie Blum, Rebekah Epstein, Stephen Watts, Anna Thalacker-Mercer

**Affiliations:** ^1^Division of Nutritional Sciences, Cornell University, Ithaca, NY, United States; ^2^Department of Biology, University of Alabama at Birmingham, Birmingham, AL, United States; ^3^Nutrition Obesity Research Center, University of Alabama at Birmingham, Birmingham, AL, United States; ^4^Department of Cell, Developmental and Integrative Biology, University of Alabama at Birmingham, Birmingham, AL, United States; ^5^UAB Center for Exercise Medicine, University of Alabama at Birmingham, Birmingham, AL, United States

**Keywords:** skeletal muscle, regeneration, tissue recovery, metabolism, nutrients

## Abstract

Skeletal muscle is fundamentally important for quality of life. Deterioration of skeletal muscle, such as that observed with advancing age, chronic disease, and dystrophies, is associated with metabolic and functional decline. Muscle stem/progenitor cells promote the maintenance of skeletal muscle composition (balance of muscle mass, fat, and fibrotic tissues) and are essential for the regenerative response to skeletal muscle damage. It is increasing recognized that nutrient and metabolic determinants of stem/progenitor cell function exist and are potential therapeutic targets to improve regenerative outcomes and muscle health. This review will focus on current understanding as well as key gaps in knowledge and challenges around identifying and understanding nutrient and metabolic determinants of skeletal muscle regeneration.

## Introduction

Maintenance of skeletal muscle mass and function are essential for quality of life. Skeletal muscle is the largest organ system in the body, accounting for 30–40% of whole body mass of a healthy adult (Janssen et al., 1985). Each skeletal muscle is composed of an intricately connected network of nerves, blood vessels, and bundles of myofibers that contain myofibrils with the contractile units (sarcomeres), all covered and supported by layers of connective tissue. The contractile property of skeletal muscle allows for voluntary contractions in the body that facilitate physical locomotion, posture maintenance, breathing, urinary bladder control, mastication, swallowing, and blinking. Additionally, muscle is a major metabolic organ that sustains body temperature and plays a central role in whole body nutrient homeostasis.

Skeletal muscle repair and regeneration of damaged tissue following trauma is essential to regain tissue homeostasis. Impairments in muscle regeneration are associated with pathological tissue remodeling, which includes the gain of fat and fibrotic tissues—adverse changes that impact the organ and organismal function and metabolism. Unfortunately, existing therapies to improve regenerative outcomes are limited, and the demographic of individuals at risk for impaired muscle regeneration and poor muscle health is projected to increase in the future (Table 1). In 2016, 15% of the population was over the age of 65, by 2030 it is estimated that this will rise to 21% (Ortman et al., 2014; Medina, 2018). The current prevalence of obesity is 42% and the prevalence of overweight and obesity is 71.6% (Hales et al., 2020). According to one report that used multiple prediction models, the prevalence of obesity in 2030 is projected to range from 42 to 51% (Finkelstein et al., 2012). Further, an estimated 85% of adults will have overweight or obesity by 2030 (Wang et al., 2008). According to the National Diabetes Statistics Report, 13% of the U.S. population has diabetes (Centers for Disease Control and Prevention, 2020). According to this report, 90–95% of reported cases were type 2 diabetes. From 2015 to 2030, the proportion of adults with type 2 diabetes is projected to increase by 54% (Krohe et al., 2016). The current prevalence of chronic kidney disease is 13.2% and is projected to increase to 16.7% by 2030 (Hoerger et al., 2015).

**TABLE 1:** The prevalence of conditions known to impact muscle regeneration is going to increase.

	Recent estimate of prevalence (%)	Projected prevalence for 2030 (%)
Advanced age (>65 years) (Medina, 2018)	15	21
Obesity (Finkelstein et al., 2012; Hales et al., 2020)	42	42–51
Type 2 diabetes (Krohe et al., 2016; Centers for Disease Control and Prevention, 2020)	13	15.3
Chronic kidney disease (Hoerger et al., 2015)	13.2	16.7

Coordination of the regenerative process is multifaceted, and it is increasingly recognized that nutrient availability and cell metabolism are important determinants of muscle stem and progenitor cell function and ultimately tissue regeneration. The primary focus of this review is to discuss current knowledge in nutrient and metabolic determinants of skeletal muscle regeneration. To provide context for understanding nutrient and metabolic determinants of muscle regeneration and their importance, we will provide an overview of the regenerative process and the requirement for muscle regeneration. Further, we will briefly discuss impairments in muscle regeneration, which set the stage for the identification of therapies to improve regenerative outcomes. Finally, this review will shed light on key gaps in knowledge that limit the use of nutrient and metabolic targets as therapy to improve regenerative outcomes.

## Overview of Skeletal Muscle Regeneration

**FIGURE 1:**
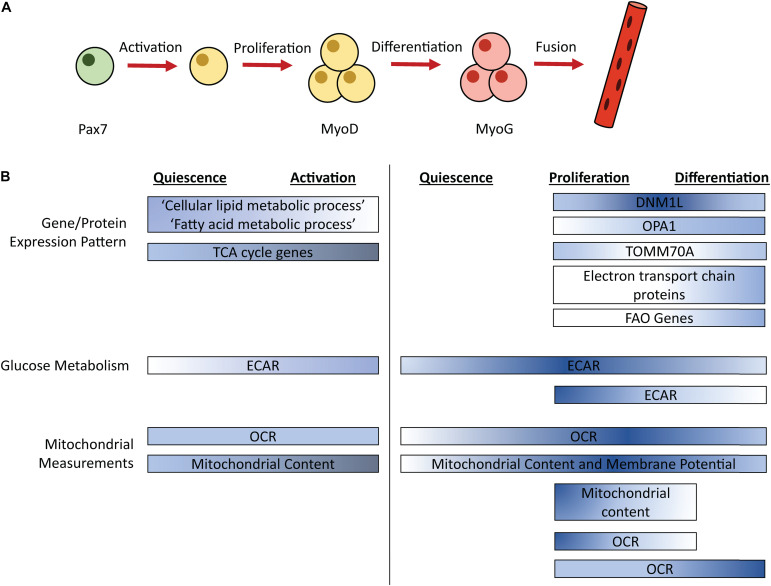
Simplified schematic of the myogenic process **(A)** with timing of metabolic pathways **(B)**. Color corresponds to relative pathway use; darker color indicates more intense relative pathway use. Comparisons are made within each bar, not between bars. ECAR, Extracellular acidification rate; OCR, Oxygen consumption rate; FAO, Fatty acid oxidation; Pax7, paired box 7; MyoD, Myoblast determination protein 1; MyoG, Myogenin.

Skeletal muscle regeneration is an obligatory process to repair damaged tissue in response to contusion, laceration, burn, and mechanical overload, enabling muscle to return to a homeostatic state. Regeneration is dependent on a well-orchestrated myogenic program that includes the activation of muscle-specific stem cells (MuSCs) and expansion of MuSCs and their committed progeny, the muscle progenitor cells (MPCs) (Figure 1A). MuSCs and MPCs will collectively be referred to as MPCs throughout, except where cell specificity is necessary. Following population expansion, MPCs undergo terminal differentiation, fuse to each other or into damaged myofibers, and undergo maturation. MuSCs generally reside in a quiescent state until activated by trauma-associated cues. Quiescent and proliferating cells are characterized by their expression of the transcription factor paired box 7 (Pax7) (Seale et al., 2000). Pax7 expression declines during terminal differentiation (Zammit, 2006). Myoblast determination protein 1 (MyoD) is an early regulator of MuSC commitment to the muscle lineage (Megeney et al., 1996). Myogenin (MyoG) is a transcription factor expressed in differentiating MPCs (Wright et al., 1989). Later stages of differentiation are characterized by the expression of embryonic myosin heavy chain (eMHC), which persists during MPC fusion, and is eventually replaced by adult myosin isoforms (Schiaffino et al., 2015).

Studies in rodents identified that MPC depletion severely impairs the muscle regeneration process, following an acute injury (Sambasivan et al., 2011; Fry et al., 2015). Additionally, MPCs are essential to prevent tissue fibrosis. Following synergist ablation surgery in mice, MPC depletion caused an increase in extracellular matrix deposition and expansion of the fibroblast cell population (Fry et al., 2014). Follow-up studies revealed that MPCs secrete exosomes containing microRNAs (specifically miR206), that can attenuate collagen biosynthesis and secretion from nearby fibroblasts (Fry et al., 2017). Thus, in response to an acute injury, robust MPC activation is required to restore muscle mass and prevent fibrosis.

The myogenic/regenerative process can be mirrored *in vitro*. For example, freshly isolated MPCs are used as model of activation. Growth media is used to promote the proliferation of MPCs. Serum withdraw (i.e., differentiation media) or allowing proliferating MPCs to become confluent, initiates differentiation of MPCs. Duration of MPC differentiation is associated with myotube formation. For example, the first day in differentiation media little to no fusion of cells is observed. However, with extended duration in differentiation media, there is an increase in the number of fused cells representing myotube formation. *In vitro* systems have been used extensively for examining nutrient and metabolic determinants of myogenesis, as detailed in the following sections.

## Impaired Muscle Regeneration and Existing Therapies

Impaired skeletal muscle regeneration is commonly observed with advancing age, and in individuals with metabolic conditions including obesity, type two diabetes, and chronic kidney disease (Chakravarthy et al., 2008; Day et al., 2010; Hu et al., 2010; Chakkalakal et al., 2012; D’Souza et al., 2013; Sousa-Victor et al., 2014; Blau et al., 2015; Avin et al., 2016; Fu et al., 2016; Liu et al., 2018; O’Sullivan et al., 2018; Teng and Huang, 2019). This impaired capacity for muscle regeneration leads to pathological tissue remodeling. Specifically, older animals or animals with obesity and insulin resistance show increased fibrotic tissue deposition and intramuscular fat deposition after injury (Brack et al., 2007; Hu et al., 2010; Lee et al., 2013). Adipose and fibrotic tissue do not perform the same essential functions as skeletal muscle (i.e., locomotion, nutrient homeostasis) and replacement of skeletal muscle with adipose or fibrotic tissue is associated with functional impairment and metabolic disease.

Impaired muscle regeneration, as observed in aging and chronic disease, is driven by a combination of changes in intracellular factors and cell-extrinsic factors. Key intracellular factors shown to be altered in conditions of impaired muscle regeneration include oxidative stress, inflammatory signaling, signal transduction, and altered metabolism (Aragno et al., 2004; Fulle et al., 2005; Price et al., 2014; Avin et al., 2016; Zhang et al., 2016; Liu et al., 2018; Pala et al., 2018). Cell-extrinsic factors include those localized to the MuSC niche (Gopinath and Rando, 2008) or circulating factors that derive from the diet or other tissues and act on MuSCs/MPCs through the bloodstream (Conboy et al., 2005), which will be discussed in the following sections.

While many therapies for improving muscle function after injury, in individuals with impairment have been proposed, as reviewed elsewhere (Baoge et al., 2012; Judson and Rossi, 2020), the overall range of options is rudimentary and there are no clinically available options. Clinical trials are planned or are already completed to test the role of cell based (MPCs and other muscle-related/resident cells) therapies to augment regeneration following a rotator cuff injury, improve muscle outcomes after hip replacement, and counteract fecal and urinary incontinence (Qazi et al., 2019). However, despite some promising results, these therapies are not yet clinically available. Identifying novel regulators of MPC function may provide new strategies to augment proposed therapies.

## Overview of Central Metabolism

**FIGURE 2:**
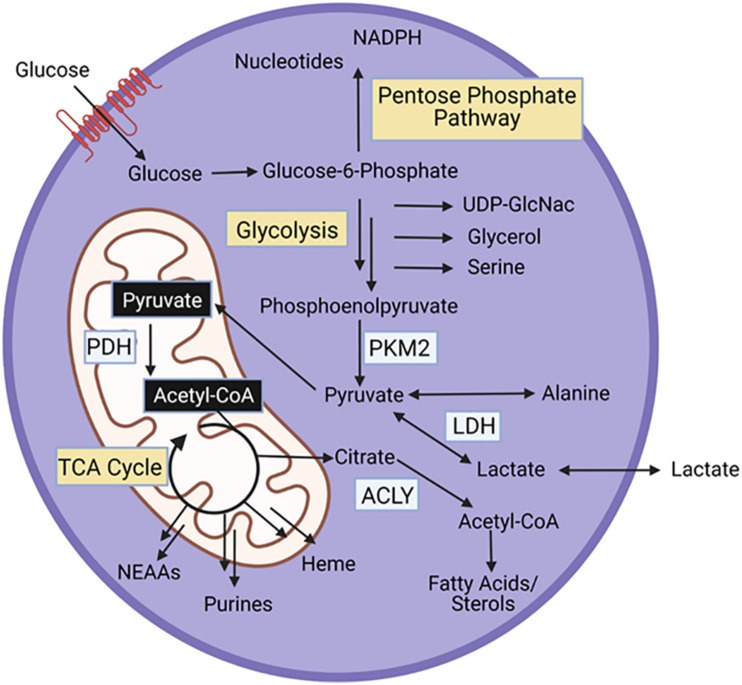
Energy generating pathways and key protein in MPCs. PKM2, Pyruvate kinase M2; PDH, pyruvate dehydrogenase; ACLY, ATP Citrate Lyase.

All cells, including muscle stem cells, require conversion of nutrients to energy and biosynthetic intermediates to support maintenance and cell division. Glycolysis, the breakdown of glucose into pyruvate, and the TCA cycle, a coordinated set of enzymatic reactions that convert acetyl-coA to carbon dioxide, are key energy generating pathways within cells (Figure 2). Acetyl-coA can be derived from pyruvate, lactate, acetate, or catabolism of fatty acids and certain amino acids (leucine, lysine, phenylalanine, tryptophan, and tyrosine). Additionally, glutamine, and other amino acids (asparagine, aspartate, valine, methionine, threonine, proline, arginine, histidine) feed the TCA cycle at entry points other than acetyl-coA. Both glycolysis and the TCA cycle generate NADH. The electron transport chain in the mitochondria, uses the energy gained from oxidizing NADH to NAD+ to power the generation of ATP. In the cytosol, lactate dehydrogenase uses NADH to reduce pyruvate to lactate, generating NAD+, which enables further glycolysis.

In addition to energy generation, glycolysis and the TCA cycle support synthesis of biosynthetic intermediates (Figure 2). Glucose-6-phosphate, the first intermediate of glycolysis is a substrate for the pentose phosphate pathway, a series of reactions that produce nucleotides, and NADPH, an essential cofactor for fatty acid synthesis and oxidative stress management. Fructose-6-phosphate, the next glycolytic intermediate is a precursor for the hexosamine pathway, which generates UDP-GlcNAc, a substrate that glycosylates proteins. Glycolytic intermediates provide a glycerol backbone for triacylglyceride synthesis, and also feed the serine biosynthetic pathway, which generates serine and glycine. Pyruvate, the final glycolytic intermediate can be transaminated to alanine. Intermediates of the TCA cycle are substrates for synthesis of fatty acids and sterols, nutritionally, non-essential amino acids (NEAAs), purines, and heme. Importantly, as intermediates in the TCA cycle are used for biosynthetic pathways, these intermediates can be replenished through anaplerotic reactions.

In the following sections we will review changes in relative occurrence of glycolytic and oxidative metabolism during myogenesis. Important to note, while these processes can be compared across the myogenic stages, the glycolytic and oxidative pathways are not mutually exclusive. Next, we will review knowledge about essentiality and function of glycolytic and oxidative metabolism, which will be combined since they are inherently linked. Following, we will briefly discuss alteration in metabolism in MPCs from populations with impaired regeneration, and then review metabolic proteins that contribute to coordination of cellular metabolism. Lastly, we will discuss historical and recent advances in understanding the role of circulating nutrients in MPC function.

## Timing of Glycolysis

Studies have examined changes in energy generating pathways that occur throughout the myogenic process (Figure 1B). Extracellular acidification rate (ECAR), a proxy measurement for glycolysis, is increased in freshly isolated MPCs (a model of activation) and proliferating MPCs compared to quiescent MPCs (Ryall et al., 2015; Pala et al., 2018). In proliferating human MPCs, mRNA levels of the glucose transporters GLUT1 and GLUT4 did not differ between an early and later timepoint of proliferation (Riddle et al., 2018). ECAR returns to levels observed in quiescent MPCs during MPC differentiation, in primary mouse MPCs isolated from injured mice (Pala et al., 2018). Similarly, in cultured primary mouse MPCs, ECAR levels are lower in differentiating, compared to proliferating, MPCs (Yucel et al., 2019). Thus, maximum ECAR occurs during MPC proliferation.

## Timing of Mitochondrial Metabolism

Similar to glycolytic metabolism, oxidative metabolism is dynamic during myogenesis. Quiescent MPCs show enrichment of expression of genes in the “cellular lipid metabolic process” and “fatty acid metabolic process” compared to activated MPCs, suggesting that lipid catabolism may be particularly important for quiescent cells (Ryall et al., 2015). Oxygen consumption rate (OCR), a measurement of mitochondrial respiration, is unaltered between quiescent and freshly isolated MPCs (Ryall et al., 2015). Interestingly, despite no change in OCR during the transition from quiescence to activation, activation is associated with an increase in mitochondrial content and expression of genes that coordinate TCA cycle activity, which may indicate increases in mitochondrial capacity precede increases in oxidative metabolism (Ryall et al., 2015). This was confirmed by a second study that showed the transition from quiescence to activation is associated with increased mitochondrial content, both in MPCs at the site of injury and in MPCs in distant muscles that also were activated by the injury (Rodgers et al., 2014). In proliferating MPCs, OCR is upregulated compared to quiescent MPCs and mitochondrial content and membrane potential remain elevated (Pala et al., 2018). Taken together, evidence suggests an increase in mitochondrial content during MPC activation that leads to increased OCR during MPC proliferation.

Oxidative metabolism is also dynamic during differentiation and myotube formation. OCR, mitochondrial content, and mitochondrial membrane potential remain elevated during MPC differentiation (5 days post-injury) compared to quiescent levels in MPCs isolated from injured mice (Pala et al., 2018). In immortalized MPCs, OCR is higher in MPCs that are differentiated for 6 days, a time when myotube formation is prevalent, compared to proliferating MPCs. Conversely, cultured primary mouse MPCs showed decreased OCR and reduced mitochondrial content in MPCs that had differentiated for 24 h compared to proliferating MPCs (Das et al., 2017; Yucel et al., 2019). Thus, it is possible that early differentiation is associated with a dip in OCR and mitochondrial content, which is recovered and perhaps magnified at later stages of differentiation.

In conjunction with elevated OCR, MPC differentiation is associated with increased fatty acid oxidation (FAO). In cultured primary mouse MPCs, only differentiated MPCs (not proliferating) show increased OCR in response to palmitate treatment, suggesting that differentiating MPCs may use more FAO compared to proliferating MPCs (Sin et al., 2016). Though interestingly, in MPCs isolated from injured mice, transcript levels of genes involved in mitochondrial and peroxisomal FAO are elevated in both proliferating and differentiating MPCs compared to quiescent MPCs (Pala et al., 2018). Thus, increases in mRNA levels of genes involved in FAO may precede increased reliance on FAO pathways. Consistent with an increase in energy demand, ATP levels were increased in both proliferating and differentiating MPCs compared to quiescent MPCs (Pala et al., 2018).

Mitochondrial morphology is also dynamic during the myogenic process. In immortalized MPCs, dynamin 1 like (DNM1L), a protein involved in mitochondrial fission, shows peak expression at 1 day differentiation compared to proliferating MPCs or MPCs differentiated for 3 or 6 days (Sin et al., 2016). OPA1, a mediator of mitochondrial fusion shows increasing expression during the differentiation process (Sin et al., 2016). Levels of translocase of outer mitochondrial membrane 70 (TOMM70A), a protein that imports mitochondrial precursor proteins, show a slight dip at the transition from proliferation to early differentiation, recovering to proliferation levels by 3–6 days in differentiation (Sin et al., 2016). Further, complexes of the electron transport chain showed increased expression after 3 days of differentiation (Sin et al., 2016). Overall, proteins involved in mitochondrial fusion and the electron transport chain increase from proliferation to differentiation.

## Essentiality of Glycolytic and Mitochondrial Metabolism During the Myogenic Process

In addition to understanding the timing of metabolic pathway enrichment, studies have addressed the essentiality of glycolytic and mitochondrial metabolism and further elucidated the specific functions that these pathways serve (e.g., energy generation, biomass production, *de novo* synthesis of metabolic intermediates and amino acids).

Glucose is essential for MPC proliferation. In the absence of glucose, EdU incorporation, a marker of cell proliferation, is strongly repressed (Chen et al., 2019). Additionally, in the absence of glucose, ATP levels are reduced, suggesting that glucose carbons are essential for energy generation in proliferating MPCs (Chen et al., 2019). Interestingly, in one study, glucose deprivation in proliferating MPCs increased basal OCR, but dramatically reduced maximal (stress-induced) respiration (Yucel et al., 2019). This could indicate that loss of glucose increases basal OCR to compensate for loss of glycolytic ATP production. Additionally, since maximal respiration was reduced in the absence of glucose, glucose might also contribute substantially to mitochondrial metabolism under stressed conditions. In support of this claim, knockout of pyruvate dehydrogenase (PDH), the enzyme that converts pyruvate to acetyl-CoA, and thus links glycolysis to the TCA cycle, blunts MPC proliferation and decreases mitochondrial membrane potential and mitochondrial mass (Hori et al., 2019). Interestingly, the ratio of phosphorylated (Ser293)/total PDH increases during MPC proliferation (Yucel et al., 2019). Phosphorylation at Ser293 inhibits PDH activity, thus this finding indicates suppression of PDH activity during MPC proliferation. Pairing findings from these studies could suggest that PDH activity is low, but essential during MPC proliferation. PDH may be important for metabolism of pyruvate from glucose or other sources (e.g., lactate, alanine), or may have other unidentified cellular roles that are important for MPC proliferation. Together, this suggests that one reason for the essentiality of glucose during MPC proliferation is metabolism of glucose into acetyl-CoA, which is a powerful generator of NADH to fuel ATP production and continued mitochondrial metabolism. In addition to supporting mitochondrial metabolism, acetyl-coA is a substrate for histone acetylation. One tracing study, using ^13^C labeled glucose, identified that glucose contributes to histone acetylation in proliferating MPCs (Yucel et al., 2019). Another study demonstrated, using ^13^C glucose, that glucose contributes minimally to the synthesis of the non-essential amino acids, serine and glycine, in proliferating MPCs; however, the contribution of glucose to serine or glycine is not sufficient to maintain proliferation in the absence of extracellular serine and glycine (Gheller et al., 2020). The necessity of glucose for other biosynthetic pathways has received limited attention. Similar to other proliferating cells, it is likely that glucose also feeds biosynthetic pathways such as the pentose phosphate pathway, and that flux of glucose through these pathways is essential for MPC proliferation, though this has never been explicitly demonstrated.

In addition to proliferation, studies have also addressed the essentiality and specific function of metabolic pathways during differentiation. Knockout of PDH blunts MPC differentiation, highlighting the importance of mitochondrial glucose metabolism during differentiation (Hori et al., 2019). Glucose availability also regulates levels of the myogenic protein MyoD (Chen et al., 2019). In differentiating MPCs, glucose deprivation decreases basal and maximum OCR, suggesting glucose is a major substrate for mitochondrial respiration (Yucel et al., 2019). ^13^C labeled glucose is used for histone acetylation in differentiating MPCs, though the amount of ^13^C labeled glucose incorporated into histones is lower in differentiating MPCs compared to proliferating MPCs (Yucel et al., 2019).

Impairing mitochondrial metabolism impacts myogenesis. In immortalized mouse MPCs, an inhibitor of mitochondrial protein synthesis significantly impaired myotube formation, despite no change in ATP levels, suggesting a requirement for mitochondrial protein synthesis that is independent of energy demand (Hamai et al., 1997). In this model, mRNA levels of the early myogenic marker myogenin (*MyoG)* was not impacted suggesting mitochondrial protein synthesis is essential for later stages of differentiation and fusion (Hamai et al., 1997). In contrast, in immortalized quail MPCs, inhibiting mitochondrial protein synthesis reduced myogenin expression and MPC proliferation, but, similarly, reduced fusion (Rochard et al., 2000). Additionally, an ATP synthase inhibitor and mitochondrial uncoupling agent reduced myogenin levels in quail myoblasts, suggesting that mitochondrial function is essential for MPC differentiation (Rochard et al., 2000). Therefore, during differentiation, glucose is likely essential to maintain mitochondrial metabolism, and mitochondrial metabolism (from all substrates) is essential for induction of myogenic proteins and successful MPC fusion.

## Metabolism in Conditions of Impaired MPC Function

Alterations in metabolic processes have been documented in conditions of impaired muscle regeneration, most extensively in aging models. Studies demonstrate that OCR is reduced in old rodent MPCs compared to young counterparts (Zhang et al., 2016; Pala et al., 2018). This is accompanied by changes in transcript profile including decreased expression of genes in the TCA cycle, oxidative phosphorylation, and lipid metabolism (Pala et al., 2018). One study documented a corresponding increase in ECAR in MPCs from old mice (Pala et al., 2018). Interestingly, research in human primary MPCs (hMPCs) did not fully corroborate this result. While old male hMPCs had lower OCR compared to young male hMPCs, there was extensive interindividual variation among MPCs from young and old females with no age effect on OCR observed (Riddle et al., 2018). This result urges caution when directly extrapolating from mouse studies to humans and a need to consider sex differences and heterogeneity of the human population.

## Metabolic Proteins in Skeletal Muscle Regeneration

An emerging area of research is linking activity of specific metabolic proteins to control of MPC metabolism, myogenesis, and muscle regeneration. Numerous metabolic proteins that directly interconvert substrates are essential for MPC function. As described above, PDH knockout impairs several steps of the myogenic process. In mice, PDH knockout impairs regeneration after a muscle injury (Hori et al., 2019; Yucel et al., 2019). In addition to PDH, ATP citrate lyase (ACLY), the enzyme that converts cytosolic citrate to acetyl-coA regulates myogenesis. ACLY is essential for differentiation, but not proliferation, in MPCs. Further, ACLY overexpression improves muscle regeneration after injury (Das et al., 2017). Lactate dehydrogenase (LDHA), the protein that converts pyruvate to lactate is implicated in maintenance of quiescence in MPCs. Injecting muscles with a plasmid that causes LDHA overexpression, 5 days after inducing a muscle injury, caused an increase in quiescent cells per muscle fiber, suggesting LDHA plays a central role in the return of activated MPCs to the quiescent state (Theret et al., 2017). Pyruvate kinase M2, the enzyme that converts phosphoenolpyruvate to pyruvate, the final step in glycolysis, is also a regulator of myogenesis (Ryall, 2013). MPCs from mice with a whole-body PKM2 deletion show impaired proliferation *in vitro* (Lunt et al., 2015). Further, giving supplemental PKM2 to MPCs increases proliferation (Kodani et al., 2018). It is unknown whether PKM2 impacts regeneration after injury.

Proteins that deposit or remove post-translational modifications also control the regenerative process. The sirtuin family of enzymes regulate protein activity by removing acyl moiety post-translational modifications (i.e., acetyl, succinyl, malonyl groups) from lysine residues of proteins. There are seven members of the sirtuin family that differ in subcellular compartment and specificity for different acyl moieties (Carafa et al., 2016). The sirtuin family of enzymes have been shown to regulate broad cellular functions including metabolism, inflammation and DNA damage repair (Bosch-Presegue and Vaquero, 2014; Vachharajani et al., 2016). Roles for a few sirtuin enzymes have been described in MPCs. For example, the deacetylase enzyme sirtuin 1 (SIRT1) promotes MPC proliferation (Rathbone et al., 2009) and is essential for recovery from muscle injury (Ryall et al., 2015). Another member of the sirtuin family, SIRT3, regulates MPC differentiation (Khalek et al., 2014). Roles for other sirtuin enzymes, such as sirtuin 5 (SIRT5), a desuccinylase and demalonylase enzyme, have been described in cancer cell proliferation (Park et al., 2016; Xiangyun et al., 2016; Chang et al., 2018), and thus are putative regulators of muscle regeneration. Succinylation of lysine residues impacts proteins involved in FAO, branched chain amino acid catabolism, ketone body synthesis, the pentose phosphate pathway, and the TCA cycle (Rardin et al., 2013; Zhang et al., 2015; Zhou et al., 2016). Additionally, over half of glycolytic enzymes are modified by malonylation, another modification removed by SIRT5 (Nishida et al., 2015). SIRT5 has been identified as a mediator of proliferation in lung tumors, hepatocellular carcinoma, melanoma, and mouse embryonic fibroblasts (Zhou et al., 2016; Xiangyun et al., 2017; Chang et al., 2018). Overexpression of SIRT5 in HEK cells increases ECAR and SIRT5 knockout reduces lactate production in hepatocytes (de Moura et al., 2014; Nishida et al., 2015). Interestingly the opposite is observed in MDA-MB-231 cells, SIRT5 reduction increased lactate levels (Polletta et al., 2015). Together these findings suggest SIRT5 is a key regulator of glucose flux. SIRT5 may also be important for oxidative phosphorylation, as SIRT5 overexpression increased basal OCR in HEK293 cells and SIRT5 reduction reduced mitochondrial membrane potential in MPCs and MDA-MB-231 cells (de Moura et al., 2014; Polletta et al., 2015). Given the role of SIRT5 in cell proliferation and metabolism, and link of metabolism to MPC function, it is possible SIRT5 is an important mediator of MPC function. In addition to the sirtuin enzymes, AMP activated protein kinase (AMPK) is a cellular energy sensor that regulates metabolism through phosphorylating other proteins (Fu et al., 2015; Theret et al., 2017). AMPK knockout MPCs have impaired activation and proliferation, and loss of AMPK impairs regeneration after injury (Fu et al., 2015; Theret et al., 2017).

Lastly, some proteins with non-metabolic canonical functions are regulators of MPC metabolism and muscle regeneration. STAT3, an intracellular signaling protein best recognized for its role in promoting inflammation, also increases OCR in MPCs (Sala et al., 2019). MPC specific loss of STAT3 impairs proliferation and blunts regeneration after injury (Zhu et al., 2016). Ying Yang 1 (YY1) is a transcription factor with numerous targets, many involved in cell growth (Deng et al., 2010). Recently YY1 was shown to be a hypoxia inducible factor 1 (HIF1α) binding partner in MPCs that coordinates metabolic capacity (Chen et al., 2019); YY1 deletion decreases both ECAR and OCR, causing a 70% reduction in ATP production (Chen et al., 2019). YY1 is essential for MPC activation and proliferation, and deletion impairs muscle regeneration after injury (Chen et al., 2019).

Expanding access to and use of proteomics has enabled and will continue to accelerate identification of metabolic proteins that are important for MPC function. Further, immunoprecipitation coupled to proteomics should continue to be leveraged to identify protein-protein binding partners, which enables the identification of “moonlighting” effects of proteins and provides crucial information about coordination of different metabolic processes or coordination of metabolism with other cellular processes. Immunoprecipitation for post-translational modifications, coupled with proteomics, can also clarify the role of these modifications in myogenesis by identifying protein targets. In addition to generating fundamental knowledge about MPC biology, continued research investigating metabolic proteins has the potential to identify novel therapeutic targets to boost regeneration in populations with impairment.

## Nutrient Availability and Dietary Factors Impact Skeletal Muscle Regeneration and Recovery

Nutrient availability and requirements for myogenesis is also a growing area of research. Identification and characterization of nutrients that are essential for MPC function has the potential to improve skeletal muscle regeneration in individuals/populations that have impairments. Nutrient availability and dietary compounds will be discussed below.

Heterochronic parabiosis studies cemented the importance of circulating factors (i.e., hormones, nutrients, cytokines in the serum) for controlling muscle regeneration (Conboy et al., 2005). In heterochronic parabiosis, the circulatory system of a young and old mice are surgically joined. When challenged to recover from a muscle injury, old mice joined to young mice (heterochronic) showed improved regeneration compared to old mice joined to another old mouse (isochronic) (Conboy et al., 2005). Follow-up studies showed that, compared to isochronic parabiosis, young mice in heterochronic parabiosis showed lower BrdU incorporation, a marker of cell proliferation, and increased fibrotic tissue infiltration (Andrew et al., 2007). The opposite was observed in old mice, in which heterochronic parabiosis improved cell proliferation and reduced fibrosis. Interestingly, replacing a portion of old plasma with saline solution improves muscle regeneration in mice (Mehdipour et al., 2020). This suggests that factors which antagonize muscle regeneration may accumulate with age, and dilution of these factors is beneficial. Additionally, therapeutic plasma exchange (TPE), an FDA approved procedure in which a portion of a person’s plasma is replaced with saline, resulted in a plasma sample that increased proliferation of cultured MPCs compared to plasma from the same individual before TPE (Mehdipour et al., 2020).

Follow-up research has identified specific factors in circulation, such as nutrients, that are essential for muscle tissue regeneration. For example, our lab demonstrated that the NEAA L-serine and the closely related NEAA L-glycine are required from an extracellular source for human MPC proliferation in cell culture models (Gheller et al., 2020). Additionally, a diet deficient in L-serine and glycine impaired recovery from myotoxin-induced muscle injury by reducing the number of MPCs and inducing intramuscular fat infiltration in aged animals (Gheller et al., 2020). Of note, several studies have identified a decline in endogenous serine levels in humans (Pitkanen et al., 2003; Houtkooper et al., 2011; Fazelzadeh et al., 2016; Gheller et al., 2020) and a decline in endogenous glycine in mice (Houtkooper et al., 2011) with advancing age. Thus, supplementation may be required to support optimal tissue regeneration. In another study, using genome-scale metabolic modeling, Shcherbina et al. (2020) predicted reduced retinoic acid signaling in aged MPCs. They identified a reduction in retinoic acid (Vitamin A) receptor mRNA levels in MPCs from aged mice compared to MPCs from young mice. Intriguingly, in both the young and aged MPCs, treatment with all-trans-retinoic acid promoted quiescence and reduced proliferation *in vitro*. However, while all-trans-retinoic acid increased Pax7 levels in young MPCs, no difference in Pax7 levels were observed in old MPCs (Shcherbina et al., 2020). Vitamin D has been shown to improve functional recovery at 48 h and 7 days post-mechanical load induced muscle injury in young human males (Owens et al., 2015). Further, this group identified a positive relationship between circulating 25(OH)D and peak torque recovery. These studies suggest a potential benefit of dietary supplementation with nutrients in the timeframe post-skeletal muscle trauma to improve tissue regeneration and functional recovery, particularly for individuals who may have reduced nutrient availability.

The relationship between nutrient availability and MPC function highlights an opportunity for habitual diet to impact MPC function through changing circulating nutrient availability or levels of nutrient linked hormones (e.g., insulin). Yet, few studies have investigated the impact of dietary patterns or whole foods on muscle regeneration and recovery. Caloric restriction is one of the best-known interventions to extend life-expectancy across species. Young mice (5 months) fed a reduced calorie diet had an increased MPC number and an increased number of MPCs per myofiber. The MPCs from the calorie restricted mice demonstrated increased OCR and reduced ECAR compared to mice consuming the control diet (Cerletti et al., 2012). Additionally, the engraftment capacity of MPCs from calorie restricted mice or engraftment capacity into a calorie restricted mouse was significantly improved compared to control mice (Cerletti et al., 2012). Conversely, muscle regeneration was impaired in mice fed a high fat diet that induced obesity (Fu et al., 2016). In terms of whole foods, a recent study from our laboratory demonstrated blueberries alter the serum environment to provide benefits to MPCs, which may optimize muscle regeneration. Our lab developed a novel paradigm to culture isolated human MPCs in human serum. Using this model, we cultured MPCs from young and old donors in serum from before and after a 6-week dietary intervention of consuming a daily freeze-dried blueberry supplement. In young cultures, the dietary intervention serum boosted MPC proliferation (Blum et al., 2020). While results for MPC function are promising, a current limitation with using dietary patterns and whole foods is identification and understanding of specific biological components that promote regeneration.

Further investigation into circulating factors that alter MPC function will likely greatly accelerate diagnosis and treatment for impaired tissue regeneration. Metabolism is a composite of human biological information, including genetic and environmental factors (e.g., diet, physical activity, microbiome, etc.); therefore, the serum metabolome offers comprehensive knowledge of an individual (Hill et al., 2019). From a diagnostic perspective, studying the serum metabolome will identify biomarkers of individuals at risk for, or already experiencing, impaired tissue regeneration. For example, circulating metabolites (e.g., lactate, aspartate/asparagine, glutamate/glutamine, and fatty acids) were identified as biomarkers that explain the variation in physical recovery in response to hip replacement surgery (Parker et al., 2020). From a treatment perspective, identifying nutrients that are essential for MPC function, and commonly altered in groups with impairment (e.g., serine/glycine are essential for MPC proliferation and decrease in older adults/individuals with metabolic conditions) can lead to rational individual nutrient supplementation or dietary pattern recommendations after muscle trauma.

## Discussion

Skeletal muscle health is fundamentally important for overall human health. One component of skeletal muscle health is preservation of the regenerative process of muscle post-injury. However, with advancing age and chronic disease the regenerative response is disrupted. Additionally, even in healthy young individuals, the nutrient intakes and metabolic pathways that are important for optimizing tissue regeneration are largely unknown.

### Fundamental Challenges to Identify Nutrient and Metabolic Requirements for Muscle Regeneration in Humans

One of the challenges in identifying and understanding nutrient and metabolic requirements in human health and disease is heterogeneity that exists among individuals. This diversity is induced by non-modifiable variables such as age, sex, and genetics; potentially non-modifiable variables such as obesity and chronic disease; and modifiable variables such as nutritional status, exercise, and medications (Lavin et al., 2019). As such, it is difficult to assess nutritional requirements in large cohorts of humans with similar conditions including trauma. Therefore, novel methods to determine nutrient and metabolic requirements are necessary. Identifying outcome variability in response to nutrient dose, such as those described for exercise induced muscle hypertrophy (Petrella et al., 1985; Thalacker-Mercer et al., 2013; Stec et al., 2017), will help identify responders and non-responders and target therapies to those most likely to benefit. Further, high-throughput technologies that enable the identification of multiple nutrients and metabolic targets should be leveraged to study MPC biology.

### Current Research Gaps

Nutrients that are essential for tissue regeneration and homeostasis need to be defined. This gap in knowledge is critical to address for (i) a fundamental, detailed understanding of MPC biology and the regenerative process; and (ii) for the development of potentially more readily available/feasible therapies that can be implemented through the diet or with supplementation to improve regenerative outcomes post-injury. Identifying metabolites that have a meaningful impact on cell function is challenging due to the intrinsic variability in human metabolism that is governed by genetic and environmental factors in the absence of disease (Hill et al., 2019). Novel and emerging methods to study individual variation in metabolism may lead to major advances in this area. Continued research into the relationship between nutrients or dietary intervention and recovery from muscle injury may lead to therapies that can be used independently, or in conjunction with cell-based therapies, to maximize recovery for patients suffering from muscle damage.

## Conclusion

Skeletal muscle regeneration and homeostasis are important for maintaining human health; however, with advancing age, metabolic disease, and pathological conditions skeletal muscle regeneration and homeostasis are perturbed. Existing therapies to augment muscle regeneration may be inadequate for the growing population of individuals with impairment (Table 1). Additionally, nutritional and pharmaceutical approaches to improve skeletal muscle regeneration are lacking. Nutrient based therapies are necessary, not only for the improvement of skeletal muscle heath, but also for the prevention of skeletal muscle deterioration. Thus, research is needed to improve upon existing and develop better therapies for the growing population with impaired skeletal muscle regeneration.

## Author Contributions

All authors listed have made a substantial, direct and intellectual contribution to the work, and approved it for publication.

## Conflict of Interest

The authors declare that the research was conducted in the absence of any commercial or financial relationships that could be construed as a potential conflict of interest.
